# A Case of Conservatively Managed Invasive Ceruminoma and a Review of the Literature

**DOI:** 10.1155/2014/897540

**Published:** 2014-07-22

**Authors:** T. F. C. Saunders, P. Monksfield

**Affiliations:** Department of Otolaryngology, University Hospitals Birmingham, Birmingham B15 2TH, UK

## Abstract

Ceruminomas are rare tumours arising from the apocrine sweat glands of the ear canal. We present a case of a malignant ceruminoma, which was managed with local surgical excision only rather than the wider clearance more commonly undertaken with these invasive neoplasms. We present the clinical case, histological analysis, and clinical progression for this patient and review the literature on this uncommon pathology.

## 1. Introduction

Ceruminoma is a rare tumour arising from the apocrine sweat glands of the ear canal. We present a case of a malignant ceruminoma, which was managed with local surgical excision only rather than the wider clearance more commonly undertaken. We discuss the histological analysis and clinical progression for this patient and review the literature on this uncommon pathology.

## 2. Case Report

A 39-year-old man presented in 1998 with an 18-month history of left-sided nonprogressive hearing loss. He had no symptoms of discharge, pain, tinnitus, or disequilibrium and was otherwise fit and well. On examination the left ear canal was occluded with a tumour arising from the superior aspect of the external auditory canal. The lesion did not blanch on compression or appear to pulsate on examination. Pure tone audiogram showed a mild to moderate conductive hearing loss in the left ear and normal hearing in the right ear.

A Computed tomography (CT) scan of the temporal bones showed a well-defined soft-tissue mass in the external auditory canal extending into the middle ear. There was no distortion of the ossicles or facial nerve involvement. A magnetic resonance imaging (MRI) scan with gadolinium suggested that the lesion was not vascular in nature.

Surgical exploration was carried out via an endaural approach and the tumour was excised leaving a complex perforation of the tympanic membrane. There was further tumour excised from the middle ear with preservation of the ossicular chain and the tympanic membrane was grafted with a temporalis fascia underlay graft.

Postoperatively the patient's hearing improved such that he had normal hearing in the left ear with no conductive deficit. The surgically removed specimens were a 1 × 0.5 × 0.5 cm nodule from the ear canal and further fragments excised from the middle ear. All were analysed histologically. All specimens contained poorly differentiated, most probably ceruminous carcinoma.

The patient was discussed at the joint clinicopathological multidisciplinary meeting and it was decided that the best clinical course of action would be a more radical surgical resection to clear the middle ear. This approach would likely cause further hearing loss.

The patient declined any further surgical treatment and requested active observation (1999). He was followed up at regular intervals both clinically and radiologically. At the last review he was asymptomatic and disease free clinically and radiologically on MRI scan.

## 3. Histological Analysis

The tumour showed a discohesive growth pattern comprising pleomorphic epithelioid cells possessing eccentrically located round or angulated nuclei with plentiful eosinophilic cytoplasm. “Indian file” configuration with nuclear moulding was present as were primitive trabecula and sparse tubuloglandular structures, in areas with associated stromal induction. Infrequent monomorphous solid islands exhibiting a few cribriform gaps indicate a possible in situ component.

The cytoplasm was faintly positive with diastase periodic acid-Schiff (DPAS) staining and negative with Perl's stain. There was intense cytokeratin immunoreactivity with negative epithelial membrane antigen (EMA), S100 protein, and carcinoembryonic antigen (CEA). Oestrogen and progesterone receptor studies were equivocal.

This profile supports apocrinoid differentiation and, arising in the ear canal, a primary ceruminous gland tumour is most likely.

### 3.1. Literature Review

These tumours are uncommon and as such there is limited literature available on the topic and it is accepted that there is no extensive reporting by surgical pathologists available [1]. These tumours are histologically similar to hidradenomata found in other sweat glands elsewhere in the body.

Cases of ceruminoma are rare; they have been seen to represent 0.00025% of all cancer cases and in one series just 2.4% of ear neoplasms that were referred [1, 2]. A problem that also is encountered is the amount of synonyms that can be used in the literature to describe this group of neoplasm.

Ceruminous gland tumours present in a wide range of ages [2] from 21 to 92 years, with the mean age of presentation of 48 years for ceruminous adenocarcinomas [3].

The clinical course of the growth is most accurately determined by histological diagnosis as the signs and symptoms are not always correlated with the level of invasiveness [4]. Generally the tumours are thought to grow slowly with a long subclinical phase before the symptomatic phase begins [5].

In cases of invasive ceruminomas, aggressive surgery with postoperative radiotherapy is considered to be the best management due to the ability of these tumours to invade locally and destroy middle ear structures and the surrounding skull base [4, 6, 7]. Ceruminous adenomas should be treated with a wide local excision and carcinoma with a radical block resection [8, 9]. With such excisions patients with these tumours tend to have good long-term outcomes [10].

The importance of performing the right type of excision is notable, with recurrence of disease increasing the risk of mortality from 9% with no recurrence to 83% with recurrence [1, 2, 11].

These tumours have the potential to metastasise to the lungs and to other glandular structures such as the parotid [3, 12, 13], and so close follow-up of these patients is advisable.

## 4. Discussion

Ceruminoma is an outmoded description that encompasses tumours of the ceruminous glands in the external auditory meatus. The ceruminous glands are modified apocrine sweat glands found in the deep dermis located in the cartilaginous outer two-thirds of the external auditory canal. The glands secrete a watery fluid that combines with the output of the sebaceous glands to form cerumen.

There are various case reports and case series in the literature dating back a number of years, although large series are limited. In general the guidance is to regard all of these tumours as malignant, as the only marker of malignancy seems to be bony invasion. Clinical presentation in other cases is most commonly a mass in the external ear canal followed by hearing changes or facial nerve involvement. The sizes at diagnosis are extremely variable—up to 3 cm in one reported case.

Mortality and morbidity for these tumours are variable and are worsened by extensive local invasion and systemic spread. In one case series the patients who died as a result of the disease were treated patients who experienced local recurrence. There are cases where there have been pulmonary metastasis—blood-borne spread has been seen to be a feature of adenoid cystic carcinoma.

As mentioned above, hidradenomata is an accepted classification that encompasses these tumours. There are four histological subtypes—ceruminous adenoma, pleomorphic adenoma, adenoid cystic carcinoma, and ceruminous adenocarcinoma [4, 9]. See Table 1 for characteristics of these tumours.

Immunohistochemical staining typically shows cytokeratin (AE1/AE3 and LP34) 8/8 (100%), epithelial membrane antigen (EMA) 8/8 (100%), CK7 (luminal only) 6/8 (75%), CK5/6 (basal cells predominantly) 4/8 (50%), and S100 protein. Some cases have reported expression of tyrosine crystals, similar to what is seen in salivary gland tumours [14].

This is a case of a rare invasive carcinoma of the external ear canal. This particular tumour was surgically managed with a close excision margin without any radiotherapy. This is contrary to the more conventional approach, which would be a radical excision with lateral temporal bone resection and blind pit closure of the ear canal. Other case reports have advised that this excision should be followed by postoperative radiotherapy [2] to prevent local recurrence. If the inner ear is preserved, the patient will be left with a maximal conductive hearing loss.

Ceruminoma is a rare diagnosis and these tumours display varying differentiation and aggressiveness. Most authors promote wide-local excision for all lesions and further extensive excision with or without radiotherapy for the more aggressive lesions. * *There are case reports that report progression of high-grade ceruminous adenocarcinomas showing metastatic progression to local lymph nodes and to the parotid. However distant metastases are rare and local invasion is the most common form of progression.

## 5. Conclusion

This patient was treated with a local resection and a more conservative approach at the patients' request. This has proven successful; with over 10 years of follow-up there is no local or distant recurrence and he has conserved hearing in the ear affected by the tumour.

This is surprising given the fact that the histology was poorly differentiated; one would expect this to be a more aggressive lesion with a high chance of local recurrence. It is therefore possible that some of these lesions even with evidence of invasion are not as aggressive as poorly differentiated carcinomas occurring elsewhere in the body are.

Despite this good result we would still advocate a more radical approach to treatment for these lesions than was taken in this case.

## Figures and Tables

**Table 1:** Hidradenomata subtypes.

Ceruminous adenoma	Well differentiated. Lack of invasion. Possibility of local recurrence if incompletely excised.
Pleomorphic adenoma	Similar to the pleomorphic adenomas found in sweat and salivary glands. Well-demarcated tumours.
Adenoid cystic carcinoma	Similar to tumours found in salivary glands. Propensity to invade nerves and around nerves.
Ceruminous adenocarcinoma	Infiltrates soft tissue and bone.
